# Radical vs. Local Excision in Rectal Carcinoma T1N0M0: Recurrence and Mortality Rates

**DOI:** 10.7759/cureus.25433

**Published:** 2022-05-28

**Authors:** Aisha Khalid, Zaina Aloul, Hanumant Chouhan

**Affiliations:** 1 Research, Harvard Medical School, Boston, USA; 2 Cardiothoracic Surgery, The Alfred, Melbourne, AUS; 3 General Surgery, Cardiff University, Wales, GBR; 4 Colorectal Surgery, Monash Health, Melbourne, AUS

**Keywords:** t1nomo, survival, recurrence, apr, tems, localised rectal carcinoma

## Abstract

Local transanal excision of early rectal carcinoma is an appealing treatment because of its low morbidity rates and better functional results than radical resection. However, this treatment approach is controversial due to its association with local recurrence when compared to the latter. This review aims to compare the local recurrence and mortality rates of local vs. radical excision in patients with T1N0M0 rectal carcinoma, based on data in the literature in the last 20 years. A PubMed, Cochrane, and Google Scholar search of published literature in the last 20 years was performed. A total of 12 studies were identified. Three were prospective, one was a population-based propensity matching study, one was a nationwide cohort study, one was a meta-analysis, and the remaining studies were retrospective/observational.

The mean local recurrence rate within five years from the studies selected for local excision (LE: 12.8%) was nearly double that of radical excision (RAD: 5.0%). The five-year mean survival rate for both LE and RAD groups from the studies selected was 86%, which was equal for both groups. The main predictors of poor outcomes were older age and the presence of two or more comorbid conditions.

There is a consensus amongst studies that LE is associated with inferior oncological outcomes such as postoperative complications and recurrence when compared to RAD. The higher local recurrence rates in LE are attributed to occult lymph node disease and inadequate adjunctive therapy due to suboptimal staging. There is no difference in the five-year survival rate when compared to RAD. A longer follow-up period is needed to determine whether the survival rates diverge after five years.

## Introduction and background

Colorectal carcinoma has the third highest mortality among cancers worldwide [1]. Approximately 10% of patients present with stage 1 disease (i.e., no nodal or distant metastasis), which is associated with a 90% five-year survival rate [2]. Standard workup associated with rectal carcinoma includes a digital rectal exam, endoscopic ultrasound (EUS) to assess the depth of tumour invasion into the rectal wall, colonoscopy to rule out the presence of a synchronous colonic lesion, and MRI pelvis to detect the potential lymph node metastasis [3]. Radical resection (RAD) for rectal cancer, which involves abdominal perineal resection, Hartmann resection, and low anterior resection, is the conventional treatment option. It has been linked to significant post-operative complications, including anastomotic leak, pelvic sepsis, and faecal incontinence [4]. An alternative option for patients with T1N0M0 rectal cancer is local excision (LE), including transanal excision (TE) and transanal endoscopic microsurgery (TEM). The National Comprehensive Cancer Network (NCCN) rectal cancer guidelines suggest that patients with T1N0 rectal disease, well to moderately differentiated, less than 3 cm in diameter with no lymphovascular or perineal invasion, are appropriate candidates for LE [4-3]. LE is associated with a shorter hospital stay and a faster return to routine activities [5]. LE has been offered to individuals with higher comorbidities and advanced age. However, multiple studies have reported inferior oncological outcomes, especially higher recurrence rates associated with LE [5-3]. This is partly attributed to the transanal approach being less suited to excise distal lower one-third tumors, size >3 cm, and sample draining lymph nodes [5-3]. One study stated that local excision alone in patients with T1 disease is related to a 9.7% local recurrence rate [6]. This calls into question whether the local resection of T1N0M0 rectal carcinoma is superior to RAD. This review aims to evaluate the literature on the outcomes of both these approaches for T1N0M0, with a specific focus on local recurrence rates and mortality. 

Criteria for selection and search methods 

**Figure 1:**
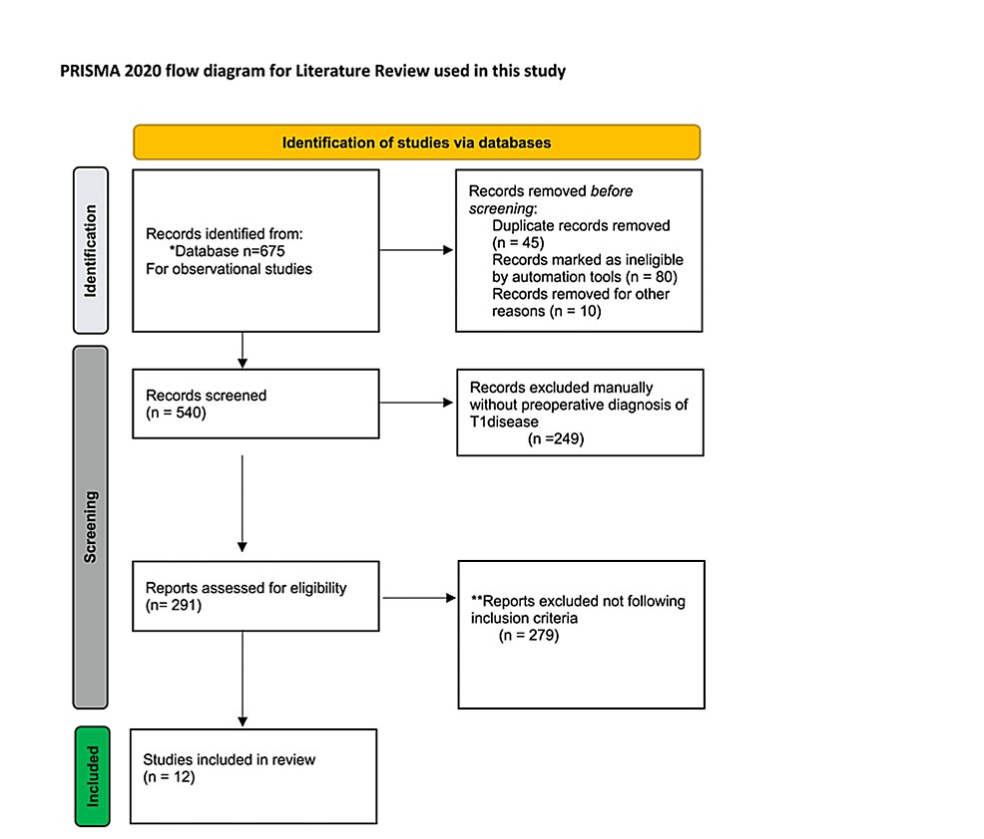
PRISMA 2020 Flow diagram for the selection of observational studies for the literature review *Google Scholar, PubMed database, Cochrane ** Inclusion criteria: Adult patients age >18 years old, with documented clinical diagnosis of T1N0M0. Preoperatively, studies at least one of the following outcomes: posteoperative mortality rates (this was the five-year survival rate for most studies), local recurrence (disease free survival rates), postoperative complication rates such as the need for permanent stoma. Studies done in last 20 years and have statistical power of 80% at least Credit: http://www.prisma-statement.org/BMJ2021 The flow diagram was used in this study via http://www.prisma-statement.org/

For this review, studies looking at T1N0M0 rectal disease were considered. Studies published more than 20 years ago were excluded, as these older studies do not accurately reflect current clinical practice. All included studies compared radical with local resection, including transanal procedures such as TE and TEM. Endoscopic polypectomies were not considered local resection methods and were excluded as such. Studies must have included adult patients aged >18 years old with a documented clinical diagnosis of T1N0M0 preoperatively. If studies considered stage 1 disease (i.e., T1 and T2 carcinomas), they were considered only if data were stratified to allow for the easy extraction of data on T1 disease. Studies had to include at least one of the following outcomes: post-operative mortality rates (this was the five-year survival rate for most studies), local recurrence, and post-operative complication rates such as the need for a permanent stoma. More than 35 patients (minimum 80% study power) were required to be present in each of the study arms to be eligible for inclusion. This was to reduce the confounders and likelihood of type II errors associated with a small sample size. Finally, although the studies used in this review were published within the last 20 years, they were only included if they analyzed data from 1980 to the present because the TEM approach became widespread after that date. We searched Cochrane, PubMed database, Google Scholar, and forward and backward citations for studies published between database inception up until 1^st^ March 2022. Two primary authors (AK, ZA) independently screened the literature search results and full-text articles to determine the final chosen publications, mainly observational studies for the review, as shown in Figure 1. The risk of bias assessment was done by using the Modified Newcastle-Ottawa quality assessment scale. All data were analyzed using Microsoft Excel 2022 (Redmond, WA) using a T-test. Most studies used Kaplan-Meier (Log-Rank) test, and results are mentioned from the pooled data. 

## Review

Results and discussion

A total of 12 studies were finalized and assessed, as shown in Table 1. When considering the local recurrence rates for LE and RAD, the risk of local cancer recurrence was significantly higher in the LE group compared to the RAD. The mean local recurrence rate within five years from the studies selected for LE was 12.8% (CI 95% 0.05-0.19), compared to only 5% (CI 95%0.03-0.07) for RAD, as shown in Tables 1, 2. This difference is not surprising given that the LE approach does not include regional lymphadenectomy. One retrospective cohort study reported that 18% of patients undergoing RAD in the population studied had at least one lymph node metastasis in the specimen resected [7]. A similar rate of occult spread is speculated in the LE group. This demonstrates that RAD provides better local control for early rectal cancers due to its superior staging in identifying patients with positive lymph nodes. This can better determine whether patients will need adjunctive therapy, unlike in the LE group, where there is no definitive method to determine the need for this treatment if patients truly need it. These findings were further supported by a prospective cohort study that looked at 40 patients undergoing LE for T1 rectal cancer. Of these, two patients developed local recurrence. Histological analysis of the specimens showed that both patients had deeper infiltration of the first third of the submucosa (sm2/3), which seems to be an additional high-risk factor for recurrence [8]. 

**Table 1:** A summary of the studies included in the literature review

Study author	Study type	Year of publication	No. of participants LE	No. of participants RAD	Survival rate LE (5 year)	Survival rate RAD (5 year)	Recurrence Rate LE (at 5 years)	Recurrence rate RAD (at 5 years)
Bentrem et al [7]	Retrospective Cohort Study	2005	151	168	93%	97%	23%	6%
Schafer et al [8]	Observational study	2005	40				5%	
You et al [9]	Retrospective cohort Study	2007	1359	765	77.40%	81.70%	13%	6.90%
Endreseth et al [10]	Prospective Cohort Study	2006	35	256	70%	80%	12%	6%
Kidane et al [11]	Systemic review and meta-analysis	2015	1274	1528	77.50%	85%	7.90%	3.40%
Celen et al [12]	Observational Study	2004	85	/	88%		12%	
Sun et al [13]	Retrospective Cohort Study	2020	/	102			29%	2%
Madbouly et al [14]	Observational Study	2005	52	/	89%		29.00%	
Cao et al [15]	Population based propensity matching study	2018	573	1146	96.60%	98.40%		
Lee et al [16]	Retrospective cohort study	2003	52	17	100%	92.90%	4.10%	0%
Oh et al [17]	Retrospective Cohort Study	2021	78	442			8.90%	9.70%
Hahnloser et al [18]	Retrospective Cohort Study	2005	77	78	89%	73%	8%	5%

**Table 2:** Analysis of LE vs. RAD local recurrence and survival rates "The decimals are being co-presented in percentages to standardize with the text. * count means number of studies examined LE: local excision, RAD: radical excision

Survival rates LE			Survival rates RAD			Local recurrence rates LE			Local recurrence rates RAD		
"Mean	0.85|85%		Mean	0.85|85%		Mean	0.12|12%		Mean	0.05|5%	
^"^Median	0.89|89%		Median	0.81|81%		Median	0.10|10%		Median	0.06|6%	
Kurtosis	0.48		Kurtosis	-2.45		Kurtosis	0.22		Kurtosis	0.01	
Skewness	-1.26		Skewness	0.17		Skewness	1.11		Skewness	-0.39	
^*^Count	9		Count	7		Count	11		Count	8	
Lower Limit	0.78		Lower Limit	0.72		Lower Limit	0.05		Lower Limit	0.03	
Upper Limit:	0.92		Upper Limit:	0.98		Upper Limit	0.19		Upper Limit	0.07	
Confidence Level (95.0%)	0.07		Confidence Level (95.0%)	0.13		Confidence Level (95.0%)	0.07		Confidence Level (95.0%)	0.02	

The five-year mean survival rate for both LE and RAD groups from the selected studies was 86%, which was equal for both groups. For LE, this ranged from 70%-93%, and for RAD, this ranged from 73%-97%; as shown in Table 1, the Kaplan Meier method was used in the studies to estimate survival. Based on these results, one could argue that LE does not pose a significant compromise compared to aggressive surgical salvage used in RAD. A nationwide cohort study reported that the type of surgery did not influence the overall survival during the five-year follow-up period, and the main predictors of poor outcomes were age 75 years and the presence of two or more comorbid conditions [9]. Covariates such as sex, ethnicity, socioeconomic status, and tumor histological grade were not statistically significant in determining the five-year survival rate [10]. 

Despite this, some studies suggested that the cancer cure rates in the LE group are less durable compared to the RAD group and that survival rates diverge after five years [11-3]. Hence, a longer follow-up period is needed to address the durability of salvage surgery and its cure rates. One randomized control trial and a meta-analysis involving 12 observational studies of 2855 patients showed that LE was associated with a statistically significant reduction in overall survival rates, with 72 more deaths per 1000 patients [11]. This may be explained by tumor location. LE was used in tumors located in the lower third of the rectum, which tend to have a poorer prognosis due to a difference in cancer biology. These findings excluded the transanal endoscopic microsurgery subgroup, which did not have lower survival rates compared to the RAD group, and are supported by a retrospective study that reported that independent risk factors for local recurrence were tumor differentiation and peri-tumoral vascular invasion, and the only risk factor for overall survival was tumor differentiation [12].

Limitations 

This review could not clarify the exact anatomical location of rectal carcinoma when LE was performed in a few studies, and the confounder for age was not addressed. 

## Conclusions

Management of T1N0M0 rectal carcinoma requires a delicate balance between cure, complications, and the functional morbidities associated with radical resection. There is a consensus amongst studies that local excision in T1NOM0 rectal carcinoma is associated with a higher recurrence rate but oncologically comparable to RAD. The higher local recurrence rates in LE are attributed to occult lymph node disease and inadequate adjunctive therapy due to suboptimal staging. However, there is no difference in the five-year survival rate when compared to RAD except when the location is not in the lower third of the rectum. A longer follow-up period is needed to determine whether the survival rates diverge in LE after five years. 
